# “To be or not to be Retained … That’s the Question!” Retention, Self-esteem, Self-concept, Achievement Goals, and Grades

**DOI:** 10.3389/fpsyg.2016.01550

**Published:** 2016-10-13

**Authors:** Francisco Peixoto, Vera Monteiro, Lourdes Mata, Cristina Sanches, Joana Pipa, Leandro S. Almeida

**Affiliations:** ^1^Centro de Investigação em Educação, ISPA – Instituto UniversitárioLisbon, Portugal; ^2^Instituto de Educação, Universidade do MinhoBraga, Portugal

**Keywords:** retention, self-esteem, self-concept, achievement goals, academic achievement

## Abstract

Keeping students back in the same grade – retention – has always been a controversial issue in Education, with some defending it as a beneficial remedial practice and others arguing against its detrimental effects. This paper undertakes an analysis of this issue, focusing on the differences in student motivation and self-related variables according to their retention related status, and the interrelationship between retention and these variables. The participants were 695 students selected from two cohorts (5th and 7th graders) of a larger group of students followed over a 3-year project. The students were assigned to four groups according to their retention-related status over time: (1) students with past and recent retention; (2) students with past but no recent retention; (3) students with no past but recent retention; (4) students with no past or recent retention. Measures of achievement goal orientations, self-concept, self-esteem, importance given to school subjects and Grade Point Average (GPA) were collected for all students. Repeated measures MANCOVA analyses were carried out showing group differences in self-esteem, academic self-concept, importance attributed to academic competencies, task and avoidance orientation and academic achievement. To attain a deeper understanding of these results and to identify profiles across variables, a cluster analysis based on achievement goals was conducted and four clusters were identified. Students who were retained at the end of the school year are mainly represented in clusters with less adaptive motivational profiles and almost absent from clusters exhibiting more adaptive ones. Findings highlight that retention leaves a significant mark that remains even when students recover academic achievement and retention is in the distant past. This is reflected in the low academic self-concept as well as in the devaluation of academic competencies and in the avoidance orientation which, taken together, can undermine students’ academic adjustment and turn retention into a risk factor.

## Introduction

The organization of the school curriculum in an increasing level of complexity in terms of knowledge learned by students requires that teachers assess whether students are able or not to move to the next grade on an annual basis. Grade retention could be defined as a practice of requiring a student to repeat a particular grade when he or she doesn’t meet the academic standards of his/her current grade level. The argument underlying this remedial practice is to provide low-achieving students with an additional opportunity to improve their achievement and meet those standards (Owings and Magliaro, 1998; Lorence, 2006, 2014; Chen et al., 2010).

However, the efficacy of this practice is controversial due to contradictory research findings on the benefits vs. the harmful effects of grade retention. Some research points to the benefits of grade retention for student achievement (e.g., Allen et al., 2009; Lorence, 2014) while other research states that holding students back a year does not improve or can even be detrimental to their academic outcomes (e.g., Jimerson et al., 1997; Jimerson, 2001; Wu et al., 2008b; Chen et al., 2010; Moser et al., 2012).

This lack of consistency is mainly a result of the different justifications and forms of implementation of the practice and is also due to methodological and measurement problems and sample characteristics of the studies (Jimerson et al., 1997; Jimerson, 1999, 2001; Lorence, 2006, 2014; Allen et al., 2009). To illustrate these inconsistencies, Lorence (2014), using a sample of 38.000 students from third to tenth grades, found that students retained in third grade outperformed their classmates who had been socially promoted (i.e., those students who had failed to meet the academic standards of their grade level but still advanced to the next grade level) in later grades. Also, Allen et al. (2009) in their meta-analysis of 22 studies concluded that the effects of retention are less negative than often claimed or have a neutral impact on student achievement.

On the opposite direction, Jimerson’s (2001) meta-analysis of 20 studies using samples of students attending kindergarten to 12th grade revealed that 80% of the studies found an unfavorable effect of retention on academic and socioemotional outcomes. While in the short term retained students can show a boost in their academic achievement, in the long term this improvement tends to decrease, disappear, or even reverse when comparing these students with their socially promoted peers (Jimerson, 1999; Wu et al., 2008a; Chen et al., 2010; Moser et al., 2012).

Besides the effects of grade retention on academic achievement, grade retention has been associated with several detrimental outcomes, such as: a lowering of self-esteem (Setencich, 1994; Jimerson et al., 1997; Martin, 2011), higher rates of school dropout (Jimerson, 1999; Jimerson et al., 2002; Jimerson and Ferguson, 2007) and school absenteeism (Jimerson, 2001), increases in aggression and disruptive behaviors (Pagani et al., 2001; Jimerson and Ferguson, 2007; Inglés et al., 2015), lower cognitive growth (Hong and Raudenbush, 2005; Roderick and Nagaoka, 2005), and a lower likelihood of completing high-school and pursuing post-secondary education (Fine and Davis, 2003).

Although retention is usually seen as a consequence of low academic achievement, this is not necessarily the case (Shepard and Smith, 1986; Huang, 2014). For example it does not explain why some low-achieving students get retained while similarly low-achieving classmates get promoted (Huang, 2014). Despite the wide range of empirical research demonstrating that grade retention can be harmful for students in several outcomes it is still a current practice. In the Portuguese context, for example, official data indicates that 13,7% of students from the 1st to the 12th grades were retained in the 2012–2013 school year (CNE, 2015). Moreover, retention rates are higher between the 5th and the 12th grades (ranging from 12,5% to 19%) than during the elementary-school years (1st to 4th grades in the Portuguese school-system, which shows a retention rate of 4%).

Given these rates it is crucial to identify which children are at most risk for grade retention and to determine which factors contribute to grade retention (Davoudzadeh et al., 2015). The most frequently cited factors in the literature associated with retention can be divided into three major groups: demographic variables (e.g., gender, socioeconomic status, ethnicity, and chronological age), parental characteristics (e.g., mothers’ educational level, parental IQ, parental involvement in school) and children’s characteristics (e.g., cognitive abilities, early school readiness skills, social and emotional skills, having special needs and academic performance).

Studies have generally shown that grade retention is more likely to occur in male (e.g., Jimerson et al., 1997; Chen et al., 2010; Huang, 2014; Klapproth and Schaltz, 2015; Davoudzadeh et al., 2015), young-for-grade children (see Huang, 2014), from low socioeconomic status (e.g., Davoudzadeh et al., 2015; Klapproth and Schaltz, 2015), and being from an ethnic minority (e.g., Klapproth and Schaltz, 2015). Retention is also more likely when the students’ parental education level is low, mothers have a lower IQ (e.g., Jimerson et al., 1997; Jimerson et al., 2006) and parents are less involved in school life (e.g., Jimerson et al., 1997).

Moreover, children are more likely to repeat a grade level when they have low cognitive abilities (e.g., McCoy and Reynolds, 1999), low school readiness skills (e.g., Duncan et al., 2007; Huang, 2014; Davoudzadeh et al., 2015), poor academic performance (e.g., McCoy and Reynolds, 1999; Huang, 2014; Davoudzadeh et al., 2015), low social and emotional skills (e.g., Willson and Hughes, 2009; Winsler et al., 2012), maladaptive behavior (Sandoval, 1984), or even when they have physical characteristics (e.g., height) associated with immaturity (e.g., Huang, 2014).

Being held back a grade may constitute a rather negative psychological experience for students (Robles-Piña, 2011), affecting their self-image and their self-perception of competence and confidence, their achievement and performance, and strongly increasing the probability of school dropout (Jimerson et al., 2002). Given this, it is important to understand the relationship between grade retention and the students’ affective components of learning, such as self-efficacy beliefs, self-esteem, self-concept, values, or motivation.

Research on the relationship between grade retention and these affective components of learning is scarce and mainly examines the effects of grade retention on these variables. Overall, findings are contradictory and follow the same tendency of those obtained on the effects of retention on subsequent achievement.

On one hand, some studies report the beneficial or neutral effects of retention. For example, Ehmke et al. (2010) have found a short-time increase in student self-concept in mathematics in the year after retention. Also Bonvin et al. (2008) found that, compared to the control group, second-grade retainers showed short-term improvements in academic self-concept, and a more positive attitude toward school, although this positive effect diminished in the course of the school year. Hong and Yu (2008) in turn have not found any detectable effects of kindergarten retention on children’s self-perceived competence 2 and 4 years after being held back, while Wu et al. (2010) in a 4-year longitudinal study, found that retention in the first grade had a positive short-term effect on children’s perceived school belonging and a positive long-term effect on perceived academic self-efficacy.

On the other hand a large number of studies report the negative effects of being retained on the affective components of learning (Martin, 2011; Robles-Piña, 2011; Goos et al., 2013; Lamote et al., 2014). Goos et al. (2013) have found that first-grade repeaters seem to be behind in several psychosocial skills, for at least a part of their primary education when compared to their similarly at-risk grade-mates who got promoted. Martin (2011) has also found that grade retention is a significant negative predictor of academic self-concept and of self-esteem and these negative effects persist in follow-up analyses using a sub-sample of retained and promoted students matched by ability and gender. Additionally, Robles-Piña (2011) has found that although those adolescents who had been retained presented higher GPA, they also reported a lower self-concept and higher rates of depression. Indeed, in this study self-concept was a stronger predictor of student retention status than GPA. Although Lamote et al. (2014) found that students retained in the 8th grade had a significantly higher academic self-concept in the year of retention, this advantage disappeared over time and by Grade 12, there was no longer any significant difference between the retained and the promoted low-achieving students.

Overall, these findings highlight the stressful nature of retention and suggest that the positive short-term effects of being retained observed in some cases, tend to decrease or even disappear in the long-term. Given these results, Robles-Piña (2011) argued that retention should be revisited from the perspective of mental health outcomes and well-being perspectives, rather than solely focusing on student academic outcomes.

Throughout the research on the impact of school achievement on the affective components of learning (e.g., self-concept, motivation), the reciprocal influence of these relationships has been also highlighted (e.g., Huang, 2011). Nevertheless, empirical evidence on the interrelationship between these variables and grade retention is much more limited, almost exclusively addressing the effects of retention on students’ subsequent self-concept, self-esteem, or motivation and not its opposite. Illustrating this idea, Marsh and Craven (2006) proposed the reciprocal-effects model (REM), arguing that prior self-concept affects subsequent achievement, and prior achievement affects subsequent self-concept. Corroborating this hypothesis, Huang (2011) in a meta-analysis of 39 longitudinal studies found significant relationships between previous self-concept and subsequent academic achievement, as well as between previous academic achievement and subsequent self-concept. However, the magnitude of the correlations between self-concept and achievement varied depending on whether these studies used a global measure of self-concept or an academic/subject-specific self-concept measure (Huang, 2011). These results are in line with research in the educational field demonstrating that academic achievement is more strongly correlated to academic self-concept than with global self-concept, and that achievement in specific domains is more strongly correlated to the corresponding specific domains of self-concept.

A recent longitudinal study by Seaton et al. (2014) also confirmed the reciprocal relations between mathematics self-concept and mathematics achievement which were highly consistent over time, even when the effects of the previous time wave were controlled for. In line with the REM (e.g., Marsh and Craven, 2006), these findings lead Seaton et al. (2014) to conclude that interventions focusing on skills improvement in mathematics are necessary but, to improve mathematics performance it is also important to promote the students positive perceptions of their abilities.

Although some studies have shown the predictive effect of students’ early approaches to learning and their social and emotional skills on early grade retention (e.g., Davoudzadeh et al., 2015), the role played by student self-esteem, their academic self-concept, or even their motivational orientations in the explanation of grade retention has rarely been considered. Studies relating retention and motivation from the point of view of achievement goal theory are uncommon, despite the goals by which students guide their action when performing learning tasks are a very important indicator of their academic performance (Boekaerts, 2002).

From the point of view of achievement goal theory, when facing an academic task, students can focus either on the acquisition of knowledge and on increasing competence (mastery/learning/task orientation) or focus on the self, ability or performance relative to others (performance/ego) (Nicholls, 1984; Ames and Archer, 1988; Elliott and Dweck, 1988; Skaalvik, 1997; Pintrich, 2000). Students endorsing ego goals can focus on outperforming others (performance approach/self-enhancing ego orientation) or on avoiding negative judgments from others (performance avoidance/self-defeating ego orientation). A fourth type of goal is avoidance orientation where the focus is on doing the least possible, escaping from school work (Nicholls, 1984; Middleton and Midgley, 1997; Skaalvik, 1997; Seifert and O’Keefe, 2001).

The type of goals that the student aspires to will lead him to focus on different elements of the learning process (Darnon et al., 2006) and consequently originating different outcomes. A meta-analysis by Linnenbrink-Garcia et al. (2008) on the relationship between goal orientation and achievement indicates that approximately 50% of the studies found a significant positive correlation between mastery goals or performance-approach goals and achievement, suggesting that both type of goals can be beneficial for achievement. In the opposite direction, performance–avoidance goals have been consistently found to be negatively related with achievement and academic performance (e.g., Elliot, 1999; Korn and Elliot, 2016).

Martin (2009) has examined the influence of grade retention on high school students’ academic motivation, engagement, and performance. He found that retained students had significantly lower scores in self-efficacy, task orientation, valuing of school, persistence, enjoyment of school, class participation, school attendance and performance, and higher scores in failure avoidance and disengagement.

If student motivational orientations and self-concept influence their achievement and academic performance, it seems likely that these variables can be good predictors of grade retention as well, although research in this area is very limited. One of the few studies addressing this issue was conducted by Nascimento and Peixoto (2012). Their longitudinal study over a school year with 9th graders showed that students that were at risk of being retained presented lower levels of global self-esteem when compared with both underachievers (students with previous retention) and good achievers. Moreover, students at risk of being retained presented lower levels of academic self-concept, similar to the underachievers and significantly lower than their successful classmates. Results also revealed that students at risk of being retained showed lower levels of non-academic self-concept than their underachieving colleagues. In motivation related variables, such as the importance attributed to academic competencies, task orientation and avoidance orientation, students at risk of retention presented low scores, closer to those exhibited by underachievers.

### The Present Study

The main goal of this study is to analyze the differences in students achievement, motivation, and self-related variables according to their retention status (students with past retention and that are going to be retained again, students with past retention but that aren’t going to be retained, students that are going to be retained for the first time and successful students – without past retention and that aren’t going to be retained). Taking into consideration that retention has an impact on academic achievement, self-representations, and motivation we expected that students differentiated in terms of retention status would present differences in those variables. Following research in this area we hypothesized that these differences would appear in academic achievement, academic self-concept, task orientation, and avoidance orientation (Jimerson, 2001; Allen et al., 2009; Martin, 2009, 2011; Chen et al., 2010; Robles-Piña, 2011; Nascimento and Peixoto, 2012; Lorence, 2014).

Adopting a person-centered approach (Magnusson, 1988; von Eye and Bogat, 2006; Tuominen-Soini et al., 2012; Pulkka and Niemivirta, 2013), we anticipated that students with recent retention could present less adaptive motivational profiles.

## Materials and Methods

### Participants

Participants were 695 Portuguese students attending 12 schools in the Lisbon region. Participants were selected from two cohorts (5th and 7th graders) of a larger group of students followed over a 3-year longitudinal research project. Student ages ranged from 10 to 17 years old (*M* = 12.11, *SD* = 1.59), 48% were in 5th grade in the beginning of the project, and 50.8% were male. In terms of educational background^1^ 16.5% of students came from families in which mothers had a university education, 30.1% attended secondary education (10th to 12th grade), 25.9% attended the 3rd cycle (7th to 9th grade) and 27.5% attended the 1st or 2nd cycle of basic education (1st to 6th grade)

The students were selected if they had already experienced retention before the beginning of the research project (past retention) or if they experienced retention in 1 of the 3 years of the project (recent retention). An additional group of students was randomly selected among those who had never been retained (either in the past or recently). Therefore, participants were assigned to four groups according to their retention-related status over time: (1) students with past retention and recent retention (PR – RR, *N* = 171); (2) students with past retention but no recent retention (PR – NRR, *N* = 104); (3) students with no past retention but with recent retention (NPR – RR, *N* = 231); (4) students with no past retention and no recent retention (NPR – NRR, *N* = 189)^2^. The distribution of the participants by gender and mother’s education level for the four groups is presented in **Table 1**.

**Table 1:** Student distribution by retention status, mother’s education level and gender.

		PR – NRR	PR – RR	NPR – RR	NPR – NRR
Mother’s Education level					
	6th Grade or low	50	57	48	29
	7th to 9th grade	40	49	69	26
	10th to 12th grade	47	42	61	64
	University	15	13	33	52
Gender					
	Males	83	94	102	75
	Females	66	70	109	96

*PR – NRR, group with past but no recent retention; PR – RR, group with past and recent retention; NPR – RR, group with no past but recent retention; NPR – NRR, group with no past or recent retention.*

### Measures

#### Self-concept and Self-esteem

Self-concept and self-esteem measures were collected through the Self-concept and Self-esteem scale for Adolescents (Peixoto and Almeida, 1999, 2010, 2011) and for Pre-Adolescents (Peixoto et al., 2016). The scale for adolescents has 51 items grouped in 10 different subscales, 9 related to specific domains of self-concept and one directed toward the evaluation of global self-esteem. Each specific domain of self-concept is assessed through 5 items and global self-esteem is a 6-item measure (e.g., “Some young people do like the way they are leading their lives”) assessing the global feeling of self-worth. The items assessing the specific domains of self-concept address school competence (e.g., “Some young people understand everything that teachers teach in class”), social acceptance (e.g., “Some young people are really well accepted by their colleagues”), athletic competence (e.g., “Some young people are very good at playing any kind of sport”), physical appearance (e.g., “Some young people don’t feel very satisfied with their appearance”), romantic appeal (e.g., “Some young people easily manage to date the people they fall in love with”), behavior (e.g., “Some young people easily get into trouble with the things they do”), close relationships (e.g., “Some young people have a special friend they can share their secrets with”), verbal competence (e.g., “Some young people manage to express themselves very well”), and competence in mathematics (e.g., “Some young people manage to solve math problems very quickly”). The Self-concept and Self-esteem scale for pre-adolescents was constructed from the version for adolescents with the same item wording but excluding two dimensions (close relationships and romantic appeal). It is possible to have global measures (e.g., academic self-concept) for both scales. Subscales used in the study consisted of academic self-concept (including school competence, verbal competence and mathematics competence), non-academic self-concept (social acceptance, athletic competence, and physical appearance), and self-esteem. Cronbach’s alphas in the three moments of data collection ranged from 0.84 to 0.85 for academic self-concept, from 0.84 to 0.87 for non-academic self-concept and from 0.75 to 0.80 for self-esteem. Items were answered in a 4-point scale ranging from “*Exactly like me*” to “*Completely different from me.*” The self-concept measures were obtained by averaging the items of each dimension.

#### Importance Attributed to Academic Competencies

The importance *attributed* to academic competencies is a six-item measure taping the same dimensions of academic self-concept on the self-concept and self-esteem scale (e.g., school competence, verbal competence, and math competence). The items are similar to those of the self-concept scale but rephrased in order for the respondent to answer in terms of the importance that he/she attributes to the self-concept dimension (e.g., “Some young people think that it is important to be a good student at school” for Importance given to School Competence; “Some young people think that it is important to be a good student in Portuguese subjects” for Importance given to Verbal Competence; “Some young people don’t think it is important to achieve good grades in Mathematics” for Importance given to Competence in Mathematics). Reliability was acceptable with Cronbach’s alpha ranging from 0.78 to 0.81. Responses ranged on a 4-point Likert scale from “*Exactly like me*” to “*Completely different from me.*” The importance attributed to academic competencies was obtained by averaging the items of this scale.

#### Goal Orientations Scale

The Goal Orientations Scale (GOS; Skaalvik, 1997; Pipa et al., 2016) is a 14-item scale measuring four types of goal orientations in academic contexts: task orientation (e.g., “Some students are interested in improving their skills in school“), self-enhancing ego orientation (e.g., “Some students always try to do better than their classmates“), self-defeating ego orientation (e.g., “When a student gives a wrong answer in class is most concerned about what their classmates think about them“), and avoidance orientation (e.g., “At school some students like to do as little as possible“). Cronbach’s alpha ranging from 0.75 to 0.76 for task orientation, from 0.80 to 0.84 for self-enhancing ego orientation, from 0.83 to 0.87 for self-defeating ego orientation and from 0.72 to 0.73 to avoidance orientation. Items were answered on a 4-point scale ranging from “*Exactly like me*” to “*Completely different from me.*” To compute the different goal orientations the items of each dimension were averaged.

#### Academic Achievement

Academic achievement was collected from students’ records at the end of the 3rd term of the school year in four core subjects: Portuguese, English, Mathematics, and Natural Sciences. A single GPA value was obtained by averaging the grades in these subjects, ranging from 1 to 5.

### Procedure

The Goal Orientation Scale, Self-concept and Self-esteem Scale and student demographical information were undertaken together with the order of presentation counterbalanced. These measures were undertaken by trained research assistants during regular classes. Parental consent was obtained and students participated on a voluntary basis. Students were informed about the study objectives and confidentiality.

### Data Analysis

For those students who had been retained over the 3 years of the project variables were calculated using data of the retention year (Time 2) and of the previous year (Time 1). Among those that had not been retained during this period, for half of them variables were computed using data of the first (Time 1) and 2nd year (Time 2) and for the other half, variables were computed using data of the second (Time 1) and 3rd year (Time 2).

Repeated measures MANCOVA/ANCOVA analyses were conducted in order to analyze the differences in self-concept, self-esteem, goal orientations, and academic achievement between the four groups of students with cohort also as a factor and age and mother’s education level as co-variates.

A cluster analysis was carried out to identify profiles based on goal orientations. Clusters analyses were conducted following the methodology proposed by Hair et al. (2010) using a hierarchical followed by a non-hierarchical classification method to decide the number of clusters. Thus the analysis using Ward’s Method and the squared Euclidean distance as a measure of similarity was carried out first, followed by the analysis using *K*-means. In order to validate the clusters obtained a discriminant analysis was conducted as well as ANOVA analysis on self-related variables and achievement. Student distribution in the different profiles according to their retention status was analyzed through the Chi square test.

In MANCOVA/ANCOVA/ANOVA analyses the effect sizes were found using partial eta squared.

## Results

**Table 2** shows the means and standard deviations for achievement and self-related variables for the four groups taken into consideration: students with past but no recent retention (PR – NRR), students with past and recent retention (PR – RR), students with no past but recent retention (NPR – RR) and students with no past or recent retention (NPR – NRR). The means revealed that the groups with retention experience (PR – NRR, NPR – RR) showed lower achievement and lower self-esteem and academic self-concept, both in the year before and in the year of retention, in comparison to their peers with no retention. In relation to non-academic self-concept, those students with past retention and those with recent retention presented higher levels than the successful group (NPR – NRR). Detailed analysis on the mean values demonstrated that the group with past and recent retention presented the lowest values in self-esteem and self-concept, and this group along with the group with no past but with recent retention presented the greatest decrease and the lower values in achievement. Moreover, the results also demonstrated that the students with recent retention (PR – RR and NRB – R) presented the lowest academic self-concept.

**Table 2:** Mean and standard deviation for the four groups for achievement and self-related variables.

	Ach-T1^1^		Ach-T2^2^		SE-T1		SE-T2		ASC-T1		ASC-T2		NASC-T1		NASC-T2	
	*M*	*SD*	*M*	*SD*	*M*	*SD*	*M*	*SD*	*M*	*SD*	*M*	*SD*	*M*	*SD*	*M*	*SD*
PR – NRR	2.92	0.33	2.89	0.31	2.82	0.57	2.87	0.54	2.46	0.46	2.49	0.39	2.87	0.49	2.86	0.46
PR – RR	2.74	0.20	2.28	0.27	2.78	0.57	2.78	0.55	2.34	0.40	2.32	0.34	2.86	0.51	2.87	0.45
NPR – RR	2.73	0.32	2.36	0.24	2.82	0.63	2.83	0.57	2.42	0.42	2.33	0.37	2.88	0.51	2.86	0.50
NPR – NRR	3.58	0.67	3.51	0.70	2.95	0.62	2.98	0.61	2.78	0.49	2.76	0.48	2.83	0.51	2.84	0.50

*Ach, Achievement; SE, Self-esteem; ASC, Academic Self-concept; NASC, Non-academic self-concept; PR – NRR, group with past but no recent retention; PR – RR, group with past and recent retention; NPR – RR, group with no past but recent retention; NPR – NRR, group with no past or recent retention.*

*^1^“Time 1” applies for the year before retention in PR – RR and NPR – RR students and for the first or the 2nd year of data collection for the students in the groups PR – NRR and NPR – NR.*

*^2^“Time 2” applies for the year of retention in PR – RR and NPR – RR students; and for the 2nd or the 3rd year of data collection for the students in the groups PR – NRR and NPR – NR.*

**FIGURE 1:**
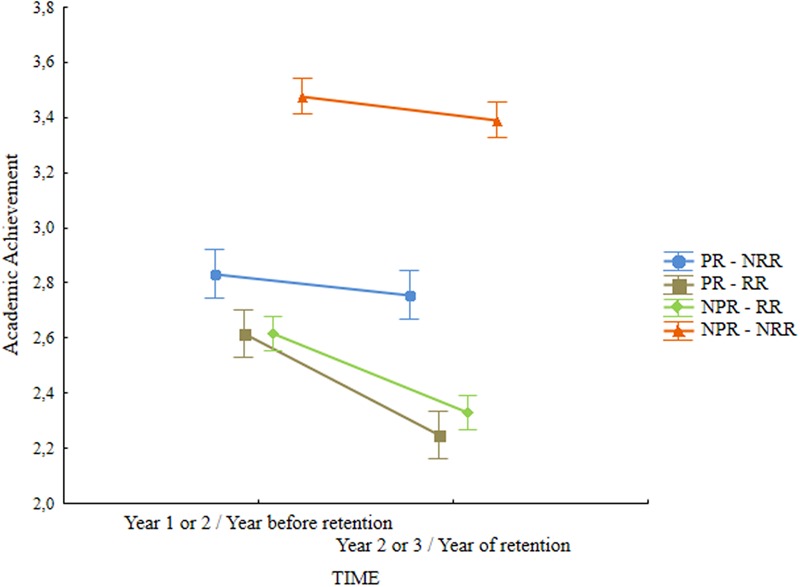
**Retention status 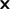 time interaction for academic achievement.** Vertical bars denote 0,95 confidence intervals. PR – NRR, group with past but no recent retention; PR – RR, group with past and recent retention; NPR – RR, group with no past but recent retention; NPR – NRR, group with no past or recent retention.

Repeated measures ANCOVA on academic achievement showed a main effect of retention status, *F*(3,525) = 174.4, *p* < 0.001, $\eta_{p}^{2}$ = 0.50, and an interaction effect between time and retention status, *F*(3,525) = 26.03, *p* < 0.001, $\eta_{p}^{2}$ = 0.13. Pairwise comparison using Bonferroni correction showed significant differences between the successful group and the other three (*p* < 0.001) as well as differences between the group of students with past but no recent retention (PR – NRR) and the two groups of students with recent retention (PR – RR and NPR – RR). Successful students showed the highest scores in academic achievement, students with recent retention (PR – RR and NPR – RR) had the lowest grades and students with past but no recent retention (PR – NRR) were in between (**Table 2**). The interaction effect between time and group was expected in the sense that the groups with recent retention (PR – RR and NPR – RR) presented a noticeable decrease in grades whereas the two groups which were not retained (PR – NRR and NPR – NRR) showed relative stability (**Figure 1**).

Repeated measures ANCOVA on self-esteem showed a small effect from retention status, *F*(3,513) = 1.54, *p* = 0.042, $\eta_{p}^{2}$ = 0.016. Pairwise comparisons with Bonferroni adjustment showed a marginal difference, *p* = 0.065, between the group of successful students (NPR – NRR) and the group of students with no past retention but with recent retention (NPR – RR). No interaction effects between time, retention status and cohort were found for self-esteem.

Repeated measures MANCOVA analysis on self-concept (academic and non-academic self-concept) showed a main effect of retention status, Pillai’s Trace = 0.219, *F*(6,1026) = 21.07, *p* < 0.001, $\eta_{p}^{2}$ = 0.110. This main effect was on academic self-concept, *F*(3,513) = 38.24, *p* < 0.001, $\eta_{p}^{2}$ = 0.183, with the successful group (NPR – NRR) presenting significantly higher academic self-concept (**Table 2**) than the other three groups (all comparisons significant at *p* < 0.001).

**FIGURE 2:**
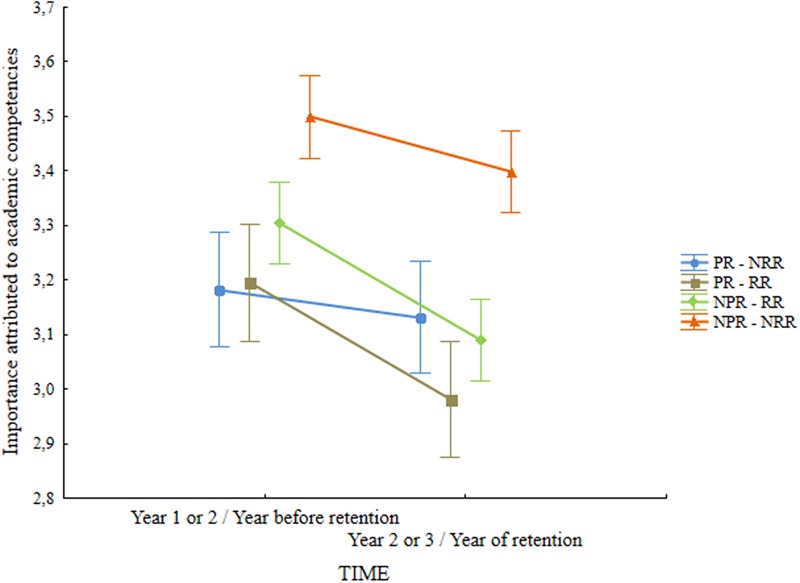
**Retention status 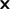 time interaction for the Importance given to academic competence.** Vertical bars denote 0,95 confidence intervals. PR – NRR, group with past but no recent retention; PR – RR, group with past and recent retention; NPR – RR, group with no past but recent retention; NPR – NRR, group with no past or recent retention.

Regarding the importance attributed to academic competencies (**Table 3**) ANCOVA analysis showed a main effect of retention status, *F*(3,513) = 11.88, *p* < 0.001, $\eta_{p}^{2}$ = 0.065, and a marginal interaction effect between time and retention status *F*(3,513) = 2.39, *p* = 0.068, $\eta_{p}^{2}$ = 0.014. Pairwise comparison between the groups showed that the successful group attributed stronger importance to academic competencies (**Table 3**) than the other three groups (*p* = 0.001 for the comparison with the PR – NRR group and *p* < 0.001 for the comparisons with the other two groups). The interaction effect between time and retention status (**Figure 2**) showed that despite the fact that in all groups the importance given to academic competencies decreased, the highest decrease was in those students with recent retention (PR – RR and NPR – RR).

**Table 3:** Mean and standard deviation for the four groups for importance given to academic competencies and goal orientations.

	IGAC-T1^1^		IGAC-T2^2^		Task-T1		Task-T2		SEnh-T1		SEnh-T2		SDef-T1		SDef-T2		Av-T1		Av-T2	
	*M*	*SD*	*M*	*SD*	*M*	*SD*	*M*	*SD*	*M*	*SD*	*M*	*SD*	*M*	*SD*	*M*	*SD*	*M*	*SD*	*M*	*SD*
PR – NRR	3.17	0.55	3.13	0.53	2.85	0.51	2.77	0.51	2.62	0.73	2.51	0.65	2.24	0.74	2.25	0.69	2.22	0.63	2.27	0.61
PR – RR	3.19	0.53	2.97	0.51	2.78	0.55	2.64	0.48	2.54	0.66	2.33	0.56	2.43	0.82	2.30	0.76	2.27	0.56	2.48	0.57
NPR – RR	3.31	0.58	3.08	0.54	2.83	0.55	2.61	0.48	2.61	0.71	2.42	0.66	2.47	0.86	2.26	0.83	2.23	0.69	2.41	0.60
NPR – NRR	3.51	0.49	3.41	0.52	3.0	0.55	2.91	0.53	2.56	0.78	2.51	0.75	2.29	0.88	2.14	0.77	2.02	0.62	2.12	0.55

*IGAC, Importance given to Academic Competencies; Task, Task orientation; SEnh, Self-enhancing ego orientation; SDef, Self-defeating ego orientation; Av, Avoidance orientation; PR – NRR, group with past but no recent retention; PR – RR, group with past and recent retention; NPR – RR, group with no past but recent retention; NPR – NRR, group with no past or recent retention.*

*^1^“Time 1” applies for the year before retention in PR – RR and NPR – RR students and for the first or the 2nd year of data collection for the students in the groups PR – NRR and NPR – NRR.*

*^2^“Time 2” applies for the year of retention in PR – RR and NPR – RR students; and for the 2nd or the 3rd year of data collection for the students in the groups PR – NRR and NPR – NRR.*

Concerning goal orientations only retention status had a small significant effect, Pillai’s Trace = 0.069, *F*(12,1536) = 3.02, *p* < 0.001, $\eta_{p}^{2}$ = 0.023. Univariate analyses showed that those effects were on task orientation, *F*(3,513) = 7.33, *p* < 0.001, $\eta_{p}^{2}$ = 0.041, and on avoidance orientation, *F*(3,513) = 7.97, *p* < 0.001, $\eta_{p}^{2}$ = 0.045. Pairwise comparison using Bonferroni correction showed significant differences between the successful group and the two groups of students with recent retention (PR – RR and NPR – RR, *p* = 0.017 and *p* < 0.001, respectively) for task orientation and between the successful group and the other three for avoidance orientation (*p* = 0.063 for the comparison with the PR – NRR group and *p* < 0.001 for the comparison with the other two groups, PR – RR and NPR – RR).

**FIGURE 3:**
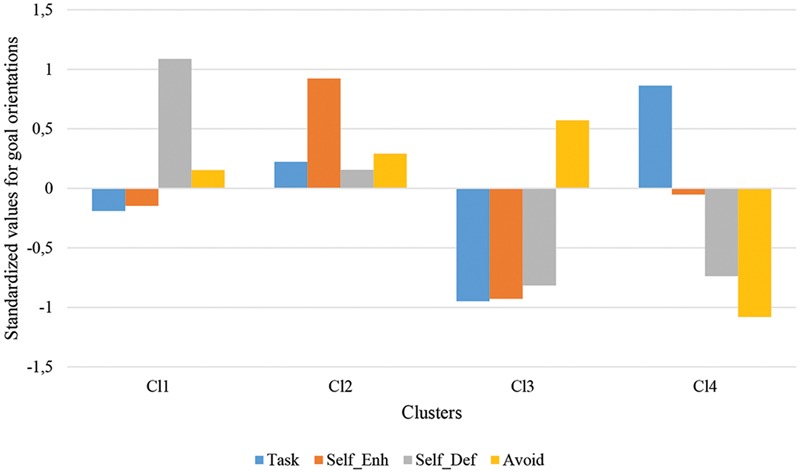
**Students’ standardized mean scores on achievement goals across the four clusters.** Task, Task orientation; Self_Enh, Self-enhancing ego orientation; Sef_Def, Self-defeating ego orientation; Avoid, Avoidance orientation.

Cluster analyses enabled to differentiate four different groups (**Figure 3**) based on students’ goal orientations in the retention year (for those who were retained) or year 2 or 3 for the others. Since the clusters obtained were very similar to those obtained in previous research (Tuominen-Soini et al., 2008, 2011, 2012; Pulkka and Niemivirta, 2013) they were given identical labels. The first cluster, labeled “self-defeating oriented,” comprised 195 students whose main characteristic was the high scores in self-defeating ego orientation. The second cluster, labeled “self-enhancing oriented,” was composed by 193 students showing high values in self-enhancing ego orientation. The third cluster was labeled “disengaged” and includes 152 students whose distinctive feature was the high scores in avoidance orientation. The fourth cluster, labeled “task oriented,” was composed by 160 students showing high scores in task orientation.

A discriminant analysis on the cluster solution revealed a 95.6% classification adequacy. ANOVA analyses on self-related variables and on academic achievement showed significant effects of the clusters, *F*(3,696) = 45.28, *p* < 0.001, $\eta_{p}^{2}$ = 0.16 for academic self-concept, *F*(3,696) = 15.05, *p* < 0.001, $\eta_{p}^{2}$ = 0.06 for non-academic self-concept, *F*(3,693) = 44.09, *p* < 0.001, $\eta_{p}^{2}$ = 0.16 for the importance accorded to academic competencies, *F*(3,694) = 21.25, *p* < 0.001, $\eta_{p}^{2}$ = 0.09, for self-esteem, and *F*(3,671) = 12.50, *p* < 0.001, $\eta_{p}^{2}$ = 0.05 for academic achievement. These results strengthened the classification reached through clusters analysis because differences found in goal orientations were also found in related variables thus supporting the validation of cluster analysis.

**Table 4** shows the composition of the clusters by the four groups of students according to their academic status (PR – NRR, PR – RR, NPR – RR, and NPR – NRR). The differences in the distribution of students according to their academic status by clusters was statistically significant, χ^2^(9) = 47.1, *p* < 0.001. Analyses through the adjusted residuals showed that successful students were underrepresented in the self-defeating and disengaged oriented cluster and prevailed in the task orientation group. Students with recent retention (NPR – RR and PR – RR) were underrepresented in the task oriented cluster and those who had been retained previously and again at the end of the year (PR – RR) were overrepresented in the disengaged group. The students with past but no recent retention were evenly distributed over the four clusters.

**Table 4:** Students distribution by retention status and clusters.

	PR – NRR	PR – RR	NPR – RR	NPR – NRR
Self-defeating oriented	24	55	76	40
Self-enhancing oriented	32	50	62	49
Disengaged	20	47	56	29
Task oriented	28	20	40	72

*PR – NRR, group with past but no recent retention; PR – RR, group with past and recent retention; NPR – RR, group with no past but recent retention; NPR – NRR, group with no past or recent retention.*

## Discussion

This study focused on analyzing the differences in academic achievement, self-related variables and motivation in students with different retention status. Results showed that a retention history and/or the perspective of being retained differentiate students both in terms of academic achievement and of the affective components of learning.

### Academic Achievement

In relation to academic achievement results showed that retention status differentiates students, with successful students (never been retained) showing the highest grades followed by students with past but no recent retentions (PR – NRR) which remain in the middle range between successful students and those who were retained at the end of the year (PR – RR and NPR – RR). Moreover, the two groups of students that were retained at the end of the school year (PR – RR and NPR – RR) showed a significant decrease in grades from the year before to the year of the retention. This interaction effect between retention status and time presented one of the strongest effects sizes, corroborating research showing previous academic achievement as one of the important predictors of retention (e.g., McCoy and Reynolds, 1999; Huang, 2014; Davoudzadeh et al., 2015). Also in line with the results from several longitudinal studies (e.g., Moser et al., 2012), even when a short term boost is observed in the grades of retained students, in the long run their grades tend to decrease and this boost tend to even dissipate over time (Jimerson, 1999; Wu et al., 2008a; Chen et al., 2010; Moser et al., 2012).

When observing and matching the trajectories of grades of these two groups of students who were retained at the end of the school year we can focus on two lines of analysis. One showing that students who are going to be retained present significantly lower grades in the year before retention. This is in line with previous findings (Huang, 2014; Davoudzadeh et al., 2015) highlighting the predictive value of school achievement in grade retention. The other, stressing that retention has no positive effect on grades, as the group with past retention also showed a decrease in grades.

These findings corroborate research showing the negative effects of grade retention in terms of academic outcomes for students who have cumulatively past and recent retentions (Martin, 2009, 2011) as well as more long term consequences that persist in young adulthood and in most cases thwart further educational achievement (Fine and Davis, 2003). However, our results can also contribute to explain differences and inconsistencies in research on the effects of retention on academic achievement. The fact of having different retention status groups and having longitudinal data allowed us to show that not all previously retained students continue to be retained. This apparently positive or neutral effect of retention for some students (Allen et al., 2009; Lorence, 2014) can be due to the different ways teachers, students and families cope with this situation, in order to promote student success (Jimerson, 2001; Brophy, 2006; Moser et al., 2012). Nevertheless, among the participants of our research only one third of retained students (37,8%) succeeded in not repeating again, showing that for the most part retention was not a positive decision. Besides academic achievement, data concerning competence beliefs and motivation also help in understanding the positive and/or harmful effects of retention for students.

### Self-representations

The effects of retention status on self-representations must be distinguished between those on global self-representations (self-esteem) and on more specific facets of self-representations (academic and non-academic self-concepts). Effects on self-esteem are minor but even then showing lower levels of self-esteem for those students who are in the path of being held back. These findings are in line with previous research showing that low achievers can exhibit low self-esteem when they forecast retention as a close possibility for their academic near future (Jimerson et al., 1997; Peixoto, 2010; Martin, 2011; Nascimento and Peixoto, 2012), as well as with research showing the absence of differences between successful students and their grade-mates with past retentions (Peixoto and Almeida, 2010). The results in the group with past retention can be explained in light of past research (Alves-Martins et al., 2002; Peixoto and Almeida, 2010), where students with past retention showed similar levels of self-esteem to their peers with no grade retention experience. These results were explained through the use of self-esteem protection mechanisms such as devaluating academic-related activities, showing negative attitudes toward school and/or presenting higher self-concepts in non-academic dimensions (Alves-Martins et al., 2002; Peixoto and Almeida, 2010).

Academic self-concept is also affected by retention status with successful students showing higher academic self-concepts than the students from the other three groups, corroborating previous research showing the effects of retention on academic self-representations (Veiga, 1995, 1996; Peixoto, 2010; Peixoto and Almeida, 2010; Martin, 2011; Robles-Piña, 2011; Nascimento and Peixoto, 2012; Rosário et al., 2013). The effect size of retention status on academic self-concept is also one of the strongest effects sizes obtained, drawing attention to the impact that retention (or the possibility of it) has on self-representation of academic competence. Moreover this result stresses that students with past but no recent retention still maintain low levels of academic self-concept even though at least 1 year mediates between the last retention and the evaluation of academic self-concept. Studies also revealed that the self-concept of students who experienced grade retention tended to decrease overtime, supporting the predictive effect of retention in self-concept (Martin, 2011; Robles-Piña, 2011; Lamote et al., 2014). However, when observing the low self-concept scores among the students of the group with no past but with recent retention, we observed that those scores were already low in the year before retention, highlighting the idea that self-concept can also predict academic achievement and retention. Overall, these data seem to be in accordance to the reciprocal effects model which maintains that self-concept is affected and also affects academic achievement (Marsh and Craven, 2006; Huang, 2011).

### Motivation

In relation to motivation both the importance attributed to academic competencies by students and their goal orientations were taken into consideration. For the importance attributed to academic competencies, results showed that successful students valued academic competencies more than the students with past and/or future retention and that the undervaluing of academic competencies is higher for those students that are going to be held back at the end of the year and for older students. Research has shown that the perception of academic competence and valuing of academic achievement have a determinant role in student behaviors such as effort and persistence (Deci and Ryan, 2000; Wigfield et al., 2012). The underlying argument is that when students believe that they are competent and value academic tasks they invest more energy and they are more persistent. Thus, affecting the value attached to academic achievement, retention affects motivation which will reflect in the attitudes of these students toward learning.

When we analyze the quality of motivation in terms of goal orientations, differences appear in task and avoidance orientations introduced by retention status. In both orientations students that are going to be retained clearly differentiate from successful students, presenting lower task orientation and higher avoidance orientation. Students with past but no recent retention did not differentiate significantly from their successful classmates in task orientation but presented higher levels of avoidance orientation. These results highlight the adaptive role of task goals and the detrimental role of avoidance goals in line with previous research (Meece et al., 2006; Nascimento and Peixoto, 2012; Federici et al., 2015). The profiles obtained through cluster analysis reinforced this finding with successful students overrepresented in the task oriented group and students with recent retention being the majority in the disengaged clusters. Students with recent retention are also predominant in self-defeating and in self-enhancing oriented clusters. If it would be expected their predominance in the disengaged and self-defeating clusters, it is a little bit surprising that they are also the majority in the self-enhancing cluster. However, observing the profile of these students shows that they also present high scores in self-defeating and avoidance orientations which is the second cluster with the highest scores in these two orientations. This result is similar to a group that Covington (2009) called “overstrivers” (with high levels in both ego/performance and avoidance orientations). According to Covington (2009) these students are simultaneously engaged in demonstrating success and, at the same time, trying to avoid failure. The result suggests that these particular students who will be retained, according to the self-worth approach, have adopted a defensive position for avoiding prospective failure by engaging in self-enhancement goals (Covington, 2009), and this may serve as a protection mechanism for further experiences of failure.

## Conclusion

The results of the present study provided additional information on the relationship between grade retention and academic achievement and its affective aspects and in the longitudinal trajectories characterizing different groups of students with different retention profiles. Four groups of students were identified according to their retention history and given the extent of the measures used in the present research our results allowed us to clarify the detrimental effect of retention both on academic and non-academic outcomes (self-concept, importance given to academic competences, motivation). Our findings also pave the way for research on a possible reciprocal effect between the retention and these affective aspects (Marsh and Craven, 2006; Huang, 2011) suggesting that retention affects and is, at the same time, affected by self-representations and the goal orientations that students pursue.

Observing the similarities between the groups with recent retention and the group with past but no recent retention and taking into consideration the longitudinal studies on the effects of retention, it is suggested that the experience of repeating a grade, whether in the past or recently and, most importantly, whether this occurred only once, leaves a profound mark on those students, undermining their achievement and socioemotional wellbeing (Jimerson, 2001). Therefore, future research should address the long-term effects of retention on socioemotional variables, by following students throughout their school career, to see whether this mark perpetuates or attenuates over time.

Despite these predominantly negative findings, the practice of grade retention continues to be a response to under-achievement in many countries. Portugal has a high rate of grade retention [more than 35% of 15 year olds had repeated one or more years, compared to an OECD average of 13,0% (OECD, 2013)]. Brophy (2006, p. 7) maintains that “low achievement patterns of grade repeaters tend to be associated with poverty indicators at both the school and the family levels.”Students in developing countries tend to repeat a grade not only because they have low achievement but also because they stop attending school the previous year. In 2015, 13,5% of Portuguese students drop out early (Ffms, 2016). Based on the assumptions of Martin (2011) other explanations can be put forward for the fact that in our country grade retention continues to be a systematic strategy used to help under-achieving students. One reason for this is because it is a direct and swift strategy to implement and doesn’t require changes in the school and school innovation. According to Brophy (2006) teachers and parents believe that repeating a year yields positive outcomes leading to such practices becoming part of the school culture.

Justino et al. (2014, pp. 90–91) argues that this culture of retention and dropout in Portugal as a solution to low achievement does not “necessarily pass for outlawing retention or to eluding the pursuit of success at any cost. The solution is before – by preventing it.” This position is shared by several researchers in different countries who affirm the need to rethink retention and its benefits as a remedial practice (Owings and Magliaro, 1998; Lorence, 2006, 2014; Chen et al., 2010). Therefore early intervention, working what students do not know, diversifying teaching methods, and engaging parents, are some preventive strategies that could be implemented to improve academic achievement (Brophy, 2006; Rebelo, 2009; Rodrigues, 2010, 2014).

## Author Contributions

FP conceived the study with input from LM and VM. CS carried out the data collection. FP conducted data analysis. FP, LM, VM, CS, and JP wrote and critically revised the manuscript. LA gave substantial contributions to the conception of the work, analysis and interpretation of the data and revised the work critically. All authors read and approved the final manuscript.

## Conflict of Interest Statement

The authors declare that the research was conducted in the absence of any commercial or financial relationships that could be construed as a potential conflict of interest.

## Footnotes

1 We used the mother’s education level as indicator of educational background because this variable is identified as highly related with student school performance (Gutman et al., 2003; Alves et al., 2016).

2 Students with recent retention are those who were retained at the end of the school year, although that information was unknown at the time the measures were completed.
