# Corrigendum: “To be or not to be Retained… That's the Question!” Retention, Self-esteem, Self-concept, Achievement Goals and Grades

**DOI:** 10.3389/fpsyg.2017.01233

**Published:** 2017-07-19

**Authors:** Francisco Peixoto, Vera Monteiro, Lourdes Mata, Cristina Sanches, Joana Pipa, Leandro S. Almeida

**Affiliations:** ^1^Centro de Investigação em Educação, ISPA - Instituto Universitário Lisbon, Portugal; ^2^Instituto de Educação, Universidade do Minho Braga, Portugal

**Keywords:** retention, self-esteem, self-concept, achievement goals, academic achievement

There is an error in the Funding statement. The statement should be “This study was supported by the FCT - Fundação para a Ciência e a Tecnologia through the research project PTDC/CPE-CED/121358/2010 and UID/CED/04853/2016.”

The authors apologize for this error and state that this does not change the scientific conclusions of the article in any way.

## Conflict of interest statement

The authors declare that the research was conducted in the absence of any commercial or financial relationships that could be construed as a potential conflict of interest.

